# Inflammation Affects the Osteogenic Differentiation of Aged Periodontal Ligament Cells via NF‐κB/FOXO3a/c‐JUN Signalling

**DOI:** 10.1111/jre.70037

**Published:** 2025-09-17

**Authors:** Luying Zhu, Zhongyuan Tang, Renjie Hu, Yaxin Li, Xuan Li, Min Gu, Yanqi Yang

**Affiliations:** ^1^ Faculty of Dentistry The University of Hong Kong Hong Kong China; ^2^ Stomatological Hospital, School of Stomatology Southern Medical University Guangzhou China

**Keywords:** ageing, inflammation, NF‐κB signalling, osteogenesis, periodontium

## Abstract

**Aim:**

This study aims to investigate the effect of inflammation on the senescence phenotype and osteogenic capacity of aged periodontal ligament cells (PDLCs), and to explore the regulatory role of the NF‐κB signalling pathway in the osteogenesis of aged PDLCs.

**Methods:**

Human PDLCs were isolated, and two ageing models were used: replicative senescence and etoposide treatment. The proliferation and migration of PDLCs were tested with the cell counting kit‐8 assay, 5‐ethynyl‐2′‐deoxyuridine staining, and scratch test. Proinflammatory cytokine levels were tested using enzyme‐linked immunosorbent assay and real time–quantitative polymerase chain reaction. Osteogenic differentiation was evaluated through alkaline phosphatase activity, Alizarin Red S staining, and calcium quantification. Expression levels of nuclear factor kappa‐B (NF‐κB) and c‐JUN pathway‐related proteins were analyzed through Western blotting.

**Results:**

Inflammatory stimulation enhanced the senescence phenotype in both young and aged PDLCs and inhibited osteogenic differentiation in aged PDLCs. During cellular ageing, NF‐κB signalling downregulated the osteogenic differentiation of PDLCs by suppressing forkhead box O3a (FOXO3a) and c‐JUN. Conversely, under exogenous inflammatory stimulation, NF‐κB signalling inhibited osteogenesis by promoting FOXO3a phosphorylation and increasing c‐JUN expression, with p21 exerting a synergistic inhibitory effect on osteogenic differentiation in aged PDLCs.

**Conclusion:**

Inflammation aggravates cellular senescence and suppresses osteogenic differentiation in aged PDLCs through the NF‐κB/FOXO3a/c‐JUN signalling pathway.

## Graphical abstract

NF‐κB/FOXO3a/c‐JUN pathway plays a crucial role in osteogenic. Differentiation of aged PDLCs in an inflammatory microenvironment, although the mechanisms of this pathway differ in cellular ageing and pathological inflammation.

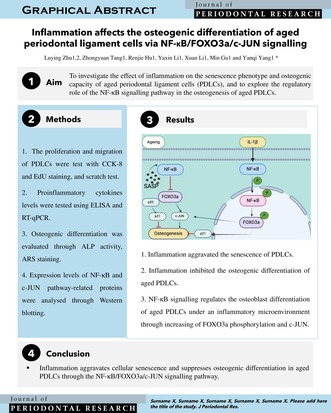

**Summary**

- Background
  ○ Ageing is a major risk factor for numerous chronic inflammatory diseases. In addition to other chronic diseases, the prevalence and severity of periodontal disease increase with ageing. PDLCs are of critical importance in the process of periodontal remodelling. However, the specific mechanisms linking inflammation and ageing in PDLCs remain unclear.
- Added value of this study
  ○ This study demonstrated that the NF‐κB/FOXO3a/c‐JUN pathway plays a crucial role in the osteogenic differentiation of aged PDLCs in an inflammatory microenvironment, although the mechanisms of this pathway differ in cellular ageing and pathological inflammation.
- Clinical implications
  ○ By elucidating the role of NF‐κB/FOXO3a/c‐JUN signalling pathway in the osteogenesis of aged PDLCs under an inflammatory environment, this study offers key insights into alveolar bone biology and periodontal metabolism, benefiting dentistry fields like periodontology. Meanwhile, this work paves the way for the development of novel therapeutic strategies, such as pharmacologic inhibition of NF‐κB phosphorylation, to enhance osteogenesis in aged patients with inflammatory diseases.

## 1 Introduction

Ageing is a time‐dependent process characterised by a progressive decline in the physiological functions and overall capacity of living organisms [1]. The prevalence and severity of periodontal disease increase with ageing, typically beginning to rise between 30 and 40 years and worsening in most adults older than 50 years [2]. In addition, moderate loss of alveolar bone and periodontal attachment is commonly observed among older adults [3]. Ageing alone does not lead to critical loss of periodontal attachment in healthy elderly persons [3]. The interplay between age‐related cellular changes (e.g., altered immune function [4]) and pre‐existing inflammation synergistically accelerates periodontal breakdown in elderly patients, particularly evident in rapid attachment loss cases [5].

Inflammation, which is frequently associated with age‐related diseases [6], exerts both detrimental and protective effects on aged cells. Inflammation can accelerate cellular senescence and tissue degeneration, thereby exacerbating age‐related diseases [7] and activating repair mechanisms that support tissue restoration [8]. For example, inflammation promotes angiogenesis, supplying oxygen and nutrients to damaged tissues [9]. Periodontal ligament cells (PDLCs), which are essential for periodontal regeneration [10], exhibit decreased viability and osteogenic differentiation with ageing [11]. Although inflammation exerts both beneficial and adverse effects on tissue repair, its specific impact on osteogenesis in the aged periodontium and the mechanisms underlying it remain unclear.

Nuclear factor kappa B (NF‐κB) signalling is a pivotal regulator of both inflammation and ageing [12]. This signalling system functions as a cytoplasmic sensor that responds to inflammatory stimuli, linking pathogenic signals to cellular responses [13]. In non‐stimulated macrophages [14] and epithelial cells [15], NF‐κB complexes are sequestered in the cytoplasm. Upon inflammatory stimulation, these complexes translocate to the nucleus, where they activate genes involved in inflammation, primarily through IκB kinases α and β (IKKs) [16]. These kinases interact with various signalling pathways and inhibit the activity of forkhead box O (FOXO) transcription factors [17]. FOXO, a major regulator of ageing, plays a crucial role in NF‐κB signalling and inflammatory responses [12]. NF‐κB activation leads to the phosphorylation of FOXO3a, thereby modulating its transcriptional activity [18].

The mammalian FOXO family comprises four members: FOXO1, FOXO3a, FOXO4, and FOXO6 [19]. These transcription factors regulate various biological processes, including development, cell signalling, tumorigenesis, and cell metabolism [20]. They also play important roles in osteoblast differentiation and skeletal homeostasis [21]. Among them, FOXO3a is particularly crucial for immunity [22], longevity [23], metabolism [24], differentiation [25], and apoptosis [26]. The activity of FOXO3a is regulated by phosphorylation, which affects its cellular localization. Nuclear localization of FOXO3a in odontoblasts increases from healthy tissue to pulpitis tissue [27]. A recent study reported that the knockdown of FOXO3a significantly reduced lipopolysaccharide‐induced inflammation, and its expression was downregulated in replicative senescent cells [28]. Abnormal expression and activation of FOXO3a are associated with age‐related bone diseases [29]. Overall, these findings indicate the essential role of FOXO3a in the processes of inflammation and ageing as well as its involvement in the regulation of the NF‐κB pathway.

FOXO3a phosphorylation is dependent on c‐JUN [18], and both proteins exhibit cross‐regulation that affects the other's gene expression [30]. c‐JUN binds to the promoter regions of FOXO3a target genes, whereas FOXO3a regulates genes encoding c‐JUN and other components of the activator protein 1 complex [31]. c‐JUN is expressed throughout osteoblast differentiation and is crucial for the early stages of gene expression during this process [32]. A study reported that c‐JUN levels are negatively correlated with bone loss in periodontitis, and its activation promotes osteoblast proliferation and differentiation [32]. Upon inflammatory stimulation, the level of c‐JUN increases, and its activation leads to the expression of inflammation‐related genes in macrophages [33]. However, whether c‐JUN plays a role in bone metabolism in the aged periodontium under inflammatory conditions has not yet been investigated.

The aim of this study is to elucidate the effect of inflammation on aged PDLCs to examine the senescence phenotype and osteogenic capacity of aged PDLCs in an inflammatory environment and to explore the regulatory role of the NF‐κB signalling pathway in the osteogenesis of aged PDLCs. The findings of this study enhance our understanding of the molecular mechanisms underlying bone metabolism in the aged periodontium under inflammatory conditions. Because oral health exerts both direct and indirect effects on overall systemic health, this study also lays the groundwork for the development of novel therapeutic strategies to support osteogenesis in aged patients with inflammatory diseases.

## 2 Methods

### 2.1 Cell Culture

Cell experiments were approved by the Hospital Authority of Hong Kong West Cluster (reference number: UW19‐140). PDLCs were isolated from the healthy teeth of young donors (*N* = 4; age = 21.25 ± 5.5 years) who provided informed consent. Periodontal health was confirmed prior to tooth extraction. Cell cultures were maintained in the alpha modification of Eagle's medium (α‐MEM) at 37°C. Two ageing models were used: the first was a replicative senescence model in which PDLCs were expanded to 15 passages, with passage (P)15 representing aged cells [34, 35], and the second model involved treating PDLCs at P3 with 10 μM etoposide (ET) [11, 36] for 24 h.

### 2.2 Exogenous Interleukin‐1β Inflammation Induction in PDLCs

Interleukin (IL)‐1β has been commonly used to induce an inflammatory microenvironment in in vitro studies [15, 37, 38, 39]. For exogenous treatment with human IL‐1β (1 ng/mL, R&D Systems, Minneapolis, MN, USA), PLDCs were seeded at 10 000 cells/cm^2^ in six‐well plates; the duration of stimulated time is 0.5 h, 24 h (acute stimulation) and 7 days, 21 days (chronic stimulation), respectively. For osteogenic differentiation assays, we adopted repeated IL‐1β stimulation for 7–21 days, consistent with established protocols [37, 38]. For immunofluorescence (IF) and enzyme‐linked immunosorbent assay (ELISA), single‐dose IL‐1β exposure was used (0.5 h for IF, 24 h for ELISA)，consistent with the methodology established by Li et al. [15, 39].

### 2.3 Colony‐Formation Assay

PDLCs were seeded at a density of 200 cells per well in 100‐mm culture dishes (Invitrogen, Carlsbad, CA, USA) for 10 days. Thereafter, the cells were fixed in 4% paraformaldehyde (PFA) for 15 min and stained with 0.1% toluidine blue (Sigma‐Aldrich, St. Louis, MO, USA) for 10 min. Excess dye was rinsed off, and colonies were observed using a stereo microscope (Olympus Optical, Tokyo, Japan). Colonies were defined as cell aggregates containing more than 50 cells.

### 2.4 Cell Proliferation Assay

Proliferation of PDLCs (P3, P7, P15, and P3 + ET) was evaluated from day 1 to day 5. PDLCs were seeded in 96‐well plates and treated with 10% cell counting kit‐8 (CCK‐8) solution in α‐MEM for 2 h at 37°C. Optical density (OD) was measured at 430 nm using a SpectraMax M2 microplate reader (Molecular Devices, Sunnyvale, CA, USA). For 5‐ethynyl‐2′‐deoxyuridine (EdU) staining, PDLCs were seeded overnight in 24‐well plates. After a 2‐h incubation with EdU (Beyotime, Wuhan, China), cells were fixed with 4% PFA and blocked using IF buffer (Beyotime). Cells were then incubated with the Click Reaction Mixture (Beyotime). Images were captured using a Nikon ECLIPSE fluorescence microscope (Nikon, Tokyo, Japan).

### 2.5 Cell Migration Assay

PDLCs were seeded overnight in six‐well plates. Upon reaching 100% confluence, a single scratch was created using a sterile 200‐μL plastic pipette tip and then the cells were cultured in medium for 24 h. Images were captured using a stereo microscope (Olympus Optical) at 0 and 24 h, and then measured by ImageJ software (National Institutes of Health).

### 2.6 Real‐Time Quantitative Polymerase Chain Reaction

Total RNA was extracted using RNeasy Cell Mini Kits (Beyotime). Real‐time quantitative polymerase chain reaction (RT‐qPCR) was performed using the StepOnePlus Real‐Time PCR System (Thermo Fisher Scientific, Waltham, MA, USA). The 2^−△△Ct^ method was used to nnormalizethe expression of target genes to that of the housekeeping gene β‐actin. Data were calculated from three biological replicates and three technical replicates. Primer sequences are listed in Table 1.

**TABLE 1:** Primers used in RT‐qPCR gene expression analysis.

Gene	Primer sequences (5′‐3′)
β‐Actin	Forward: GTGACGTTGACATCCGTAAAGA
	Reverse: GCCGGACTCATCGTACTCC
p53	Forward: CCCCTGTCATCTTTTGTCCCT
	Reverse: AGCTGGCAGAATAGCTTATTGAG
p21	Forward: CGAGAACGGTGGAACTTTGAC
	Reverse: CCAGGGCTCAGGTAGACCTT
p16	Forward: GCTCAACTACGGTGCAGATTC
	Reverse: GCACGATGTCTTGATGTCCC
IL‐6	Forward: TCCTGTCTTGCATTGCACTAAG
	Reverse: CATCCTGGTGAGTTTGGGATTC
IL‐8	Forward: ACTGAGAGTGATTGAGAGTGGAC
	Reverse: AACCCTCTGCACCCAGTTTTC
CXCL10	Forward: GTGGCATTCAAGGAGTACCTC
	Reverse: TGATGGCCTTCGATTCTGGATT
Runx2	Forward: TTCAACGATCTGAGATTTGTGGG
	Reverse: GGATGAGGAATGCGCCCTA
COLI	Forward: CTGGCGGTTCAGGTCCAAT
	Reverse: TTCCAGGCAATCCACGAGC
OCN	Forward: ATCTCACCATTCGGATGAGTCT
	Reverse: TGTAGGGACGATTGGAGTGAAA

### 2.7 Western Blotting

Cells were lysed using radioimmunoprecipitation assay buffer (Thermo Fisher Scientific). A total of 30 μL of protein was separated on a 4%–20% sodium dodecyl sulphate–polyacrylamide gel (Beyotime) and transferred to a 0.45‐μm polyvinylidene difluoride membrane. Membranes were developed using the WesternBright Quantum kit (Advansta, San Jose, CA, USA). Primary antibodies included anti‐p53 (Cat. No. 21891‐1‐AP), anti‐p21 (Cat. No. 10355‐1‐AP), and anti‐p16 (Cat. No. 10883‐1‐AP) from Proteintech (1:1000) and anti‐phospho‐IKKα/β (Cat. No. #2697), anti‐IKKα (Cat. No. #11930), anti‐phospho‐NF‐κB p65 (Cat. No. #3033), anti‐NF‐κB p65 (Cat. No. #8242), anti‐phospho‐FOXO3a (Cat. No. #9466), anti‐FOXO3a (Cat. No. #2497), anti‐c‐JUN (Cat. No. #9165), and anti‐β‐actin (Cat. No. #4967) from Cell Signalling Technology (1:1000, Danvers, MA, USA). Secondary antibodies used were mouse IgG (Cat. No. #7076) and rabbit IgG (Cat. No. #7074), both from Cell Signalling Technology (1:5000).

### 2.8 Elisa

PDLCs were seeded in six‐well plates and incubated for 2 days until they reached 80% confluence. Cells were then cultured in medium supplemented with IL‐1β (1 ng/mL) for 24 h [39]. The levels of IL‐6 (Cat. No. 430504), CXC chemokine ligand 10 (CXCL10, Cat. No. 439904), and IL‐8 (Cat. No. 431504) in the supernatants were measured using ELISA kits (BioLegend, San Diego, CA, USA). Absorbance at 450 nm was measured using a SpectraMax M2 microplate reader (Molecular Devices).

### 2.9 IF Staining

The PDLCs were stimulated with IL‐1β for 0.5 h [15]. Cells were fixed with 4% PFA, and after blocking with IF blocking buffer (Beyotime) for 1 h, cells were then incubated at 4°C with the following primary antibodies: anti‐NF‐κB p65 (Cat. No. #8242) and anti‐FOXO3a (Cat. No. #2497) from Cell Signalling Technology (1:200) and anti‐Stro‐1 (Cat. No. NBP1‐48356) and anti‐CD146 (Cat. No. NBP2‐44510) from Novus Biologicals (1:200, Centennial, CO, USA). After primary antibody incubation, cells were incubated with secondary antibodies, namely, anti‐mouse IgG (Cat. No. #8890) and anti‐rabbit IgG (Cat. No. #4412; 1:1000; Cell Signalling Technology) for 1 h at room temperature. Imaged using a Nikon ECLIPSE fluorescence inverted microscope (Nikon).

### 2.10 Alizarin Red S Staining

PDLCs were seeded in six‐well plates and cultured until they reached 80% confluence. Osteogenic differentiation was induced by supplementing the medium with 10 mmol/L β‐glycerophosphate, 50 mg/L L‐ascorbic acid, and 0.1 mol/L dexamethasone. Cells were cultured under these conditions for 21 days. After fixation with 4% PFA for 15 min, the cells were stained with 1% Alizarin Red S (ARS; Sigma‐Aldrich) for 20 min at room temperature. Staining was quantified using an osteogenesis quantitation kit (Beyotime), and absorbance was measured at 562 nm using a SpectraMax M2 microplate reader, with a standard curve used for quantification.

### 2.11 Alkaline Phosphatase Staining Assay and Quantification of Alkaline Phosphatase Activity

Alkaline phosphatase (ALP) staining was performed using a BCIP/NBT Kit (Beyotime). PDLCs were cultured until they reached 80% confluence, after which osteogenic induction medium was applied for 7 days. Cells were incubated in BCIP/NBT buffer for 10 min and subsequently examined under a stereo microscope (Olympus Optical). ALP activity was quantified using an ALP assay kit (Beyotime) after 7 days of osteogenic induction. Cells were lysed in 50 μL of lysis buffer (Beyotime). Lysates were collected by scraping the cells. The resulting supernatant was collected and analyzed at 37°C using the assay kit.

### 2.12 NF‐κB Signalling Inhibition in PDLCs

To investigate the effects of signalling inhibition, PDLCs were treated with 5 μM of the NF‐κB pathway inhibitor BAY 11‐7082 (Cell Signalling Technology) dissolved in 0.1% dimethyl sulfoxide (DMSO) as a carrier. Cells were incubated in serum‐free medium containing the inhibitor for 1 h in accordance with the manufacturer's instructions. DMSO alone was used as a vehicle control under the same conditions.

### 2.13 Transfection With a Small Interfering RNA Targeting FOXO3a

To suppress the expression of FOXO3a in PDLCs, cells were transfected with a small interfering RNA (siRNA) targeting FOXO3a (si‐FOXO3a) using Lipofectamine 3000 (Cell Signalling Technology) in accordance with the manufacturer's protocol. Transfection complexes were incubated with cells in serum‐reduced Opti‐MEM (Invitrogen) for 24 h, after which the medium was replaced with α‐MEM supplemented with 10% FBS.

### 2.14 Statistical Analysis

ImageJ software (National Institutes of Health) was used to analyze IF images and quantify staining. Comparisons between two groups were made using Student's *t* test, whereas one‐way analysis of variance (ANOVA) followed by Tukey's post hoc test was applied for multiple group comparisons in GraphPad Prism 7.0 (GraphPad, San Diego, CA, USA). Calculations were performed using Microsoft Excel. All data are presented as mean ± standard deviation, and a two‐tailed *p* value of < 0.05 was considered statistically significant.

## 3 Results

### 3.1 Colony Formation, Proliferation, and Migration of PDLCs Decreased With Ageing

**FIGURE 1:**
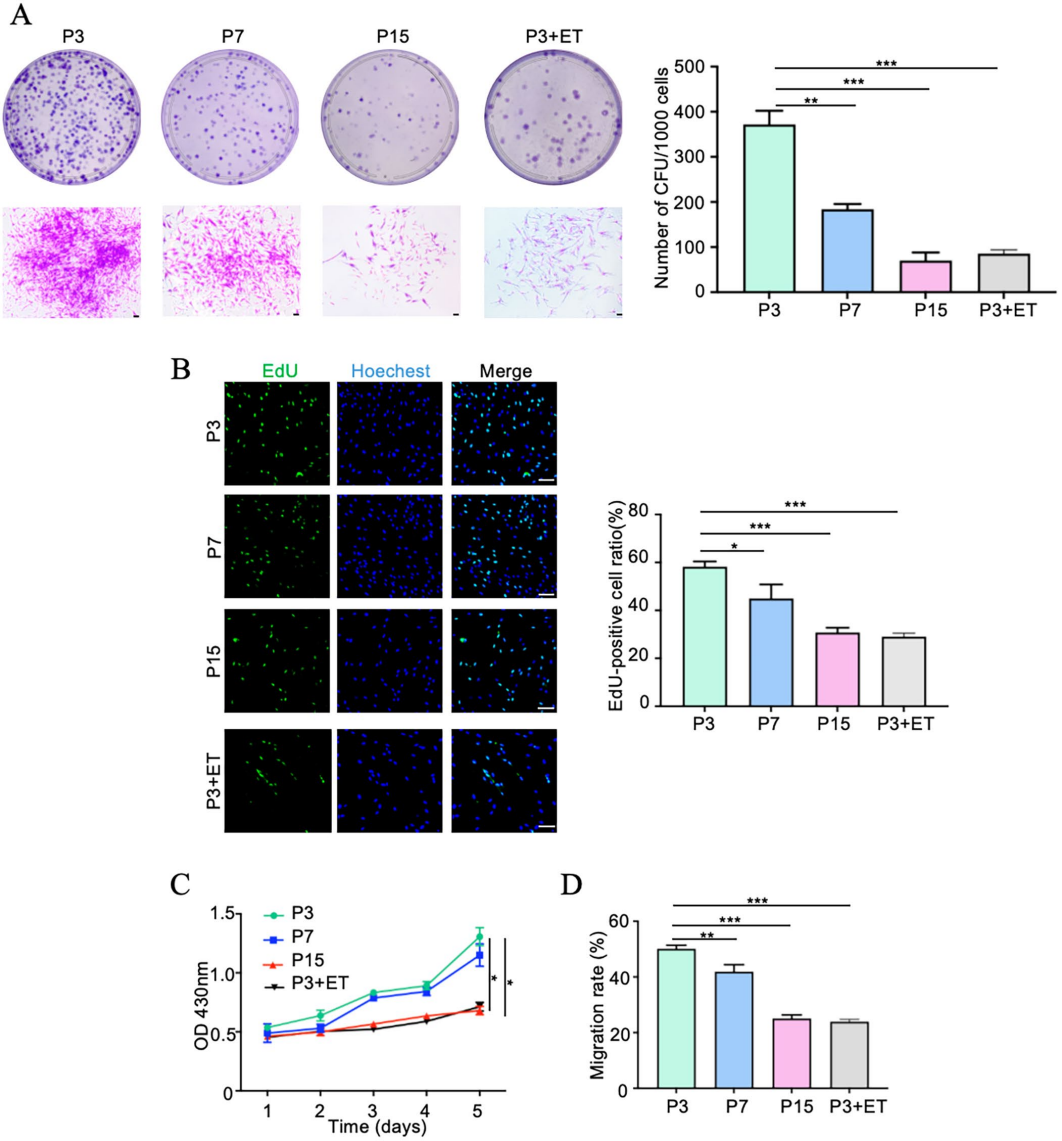
Colony formation, proliferation, and migration of PDLCs decreased with ageing. (A) The colony‐forming ability of PDLCs varied during long‐term ex vivo expansion. Representative images and quantitative analysis results are shown for colonies formed by P3, P7, P15, and P3 + ET cells, including a single representative colony from each group. (B) Cell proliferation was evaluated using the EdU assay in P3, P7, P15, and P3 + ET cells. (C) Growth curves for P3, P7, P15, and P3 + ET cells were generated using the CCK‐8 assay. (D) Cell migration in the four groups was examined over 24 h. All data were obtained from three independent experiments (*n* = 3) and the number of cell donors is three (*N* = 3). Scale bar = 100 μm; *p < 0.05, **p < 0.01, ***p < 0.001.

We used long‐term passaging in ex vivo culture and ET treatment to induce senescence in PDLCs (Figure S1). As shown in Figure 1A, PDLCs at P3, P7, and P15 and those treated with ET (P3 + ET) exhibited colony‐formation properties; however, the single‐cell colony formation rate was significantly decreased in the P15 and P3 + ET groups compared with the other groups. As shown in Figure 1B, the number of EdU‐positive cells markedly decreased in the P15 and P3 + ET groups compared with the P3 group. A similar pattern was observed in cell proliferation: P3 cells displayed the highest proliferation rate, followed by P7 cells, whereas both P15 and P3 + ET cells had the lowest proliferation rate (Figure 1C). Cell migration was evaluated using the scratch test, and the findings revealed a lower migration rate in the P15 and P3 + ET groups than in the P3 and P7 groups; the P3 group had the highest migration rate (Figure 1D). These results demonstrated that the colony formation, proliferation, and migration of PDLCs declined with ageing.

### 3.2 Inflammatory Status and Senescence Phenotype of PDLCs Were Increased During the Cellular Ageing Process and After Exogenous Inflammatory Stimulation

**FIGURE 2:**
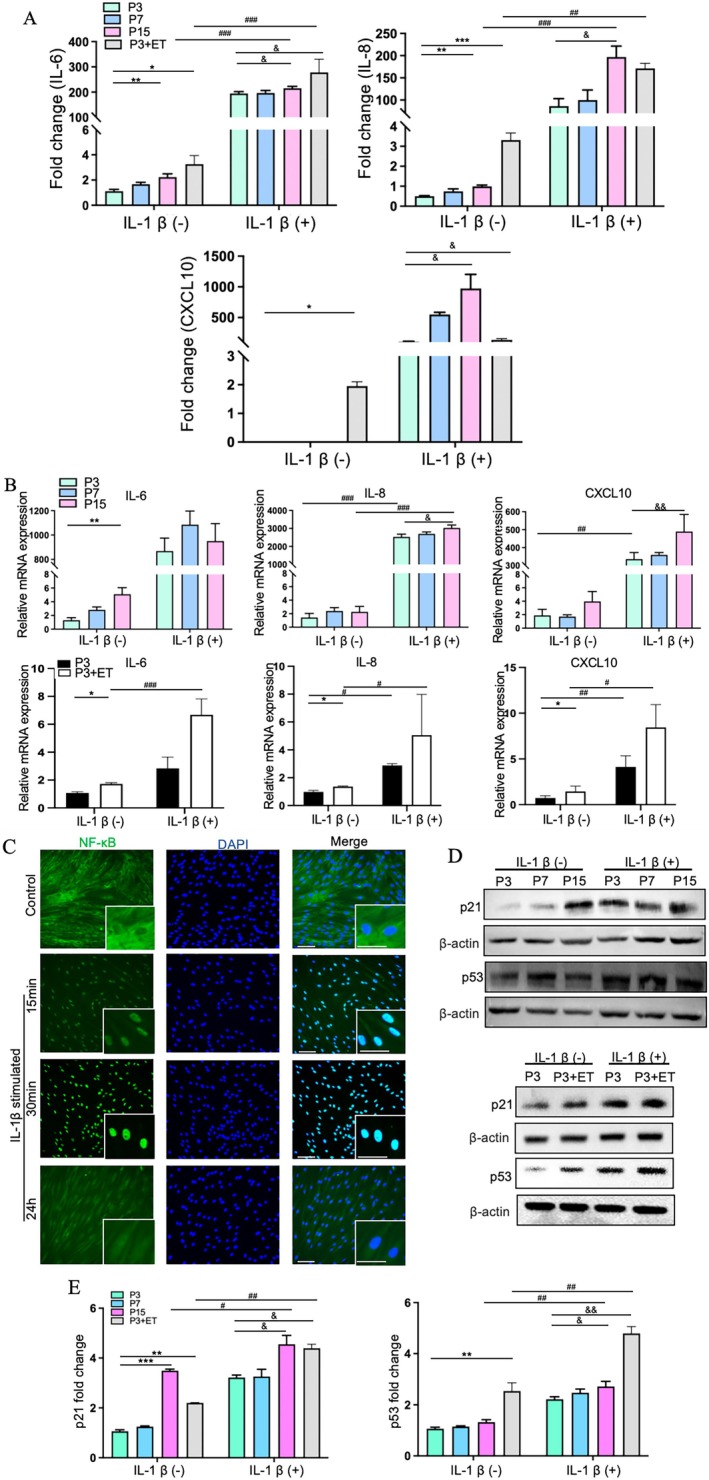
Inflammatory status and senescence phenotype of PDLCs increased during cellular ageing and after exogenous inflammatory stimulation. (A) IL‐6, IL‐8, and CXCL10 levels were quantified in P3, P7, P15, and P3 + ET PDLCs using ELISA. (B) mRNA expression of *IL‐6*, *IL‐8* and *CXCL10* in these PDLCs was measured using RT‐qPCR. (C) IF staining of NF‐κB p65 protein was conducted in PDLCs after IL‐1β stimulation at different time points (0 min, 15 min, 30 min, and 24 h). (D) Western blotting showed increased protein levels of p21 and p53 in P3, P7, P15, and P3 + ET PDLCs during ageing and after IL‐1β stimulation. (E) Quantitative analysis of Western blotting in p21 and p53. Representative data from three independent experiments (*n* = 3) and the number of cell donors is three (*N* = 3). Scale bar = 100 μm. Statistical significance is indicated as follows: * for differences between young and aged groups without inflammation (*p < 0.05, **p < 0.01, ***p < 0.001); & for differences with inflammation (^&^ *p* < 0.05, ^&&^ *p* < 0.01); and # for overall differences (^#^ *p* < 0.05, ^##^ *p* < 0.01, ^###^ *p* < 0.001).

To determine the intrinsic inflammatory status of PDLCs, we examined IL‐6, IL‐8, and CXCL10 levels in aged cells. ELISA results revealed that aged PDLCs (P15 and P3 + ET) had higher levels of these cytokines than young PDLCs (P3 and P7; Figure 2A). Similarly, the mRNA expression levels of *IL‐6*, *IL‐8*, and *CXCL10* were also increased in aged PDLCs (Figure 2B), indicating the presence of an intrinsic inflammatory state. In the absence of inflammatory stimulation, NF‐κB p65 was localised in the cytoplasm. Upon stimulation with IL‐1β, NF‐κB p65 translocated to the nucleus at 15 min, 30 min, and 24 h, with peak nuclear localisation observed at 30 min, suggesting that this time point was optimal for inducing NF‐κB‐mediated effects (Figure 2C). In Figure 2B, IL‐1β stimulation reduced *IL‐6* mRNA levels in P15 PDLCs compared to P7, though not significantly. However, P3 + ET cells showed a significant increase in IL‐6 after IL‐1β stimulation, indicating that IL‐1β enhances IL‐6 production in aged PDLCs. Meanwhile, IL‐1β stimulation further increased the expression of IL‐8 and CXCL10 in aged PDLCs, indicating an enhanced inflammatory response following exogenous stimulation (Figure 2A,B).

Expression levels of the senescence‐associated proteins p21 and p53 were higher in aged PDLCs than in young PDLCs (Figure 2D). Under control conditions, P3 + ET showed significantly higher p21 and p53 levels compared to P3. Following inflammatory stimulation, p21 and p53 expressions in P15 were moderately elevated relative to P3. In contrast, P3 + ET cells showed a more robust increase in p53 expression, accompanied by a modest but consistent elevation in p21 levels (Figure 2E). Western blotting revealed that IL‐1β stimulation increased p21 and p53 levels in both young and aged PDLCs (Figure 2D,E), indicating that inflammation triggered senescence in these cells.

### 3.3 Osteogenic Differentiation of PDLCs Decreased With Ageing and Inflammation Inhibited the Osteogenic Differentiation Ability of Aged PDLCs

**FIGURE 3:**
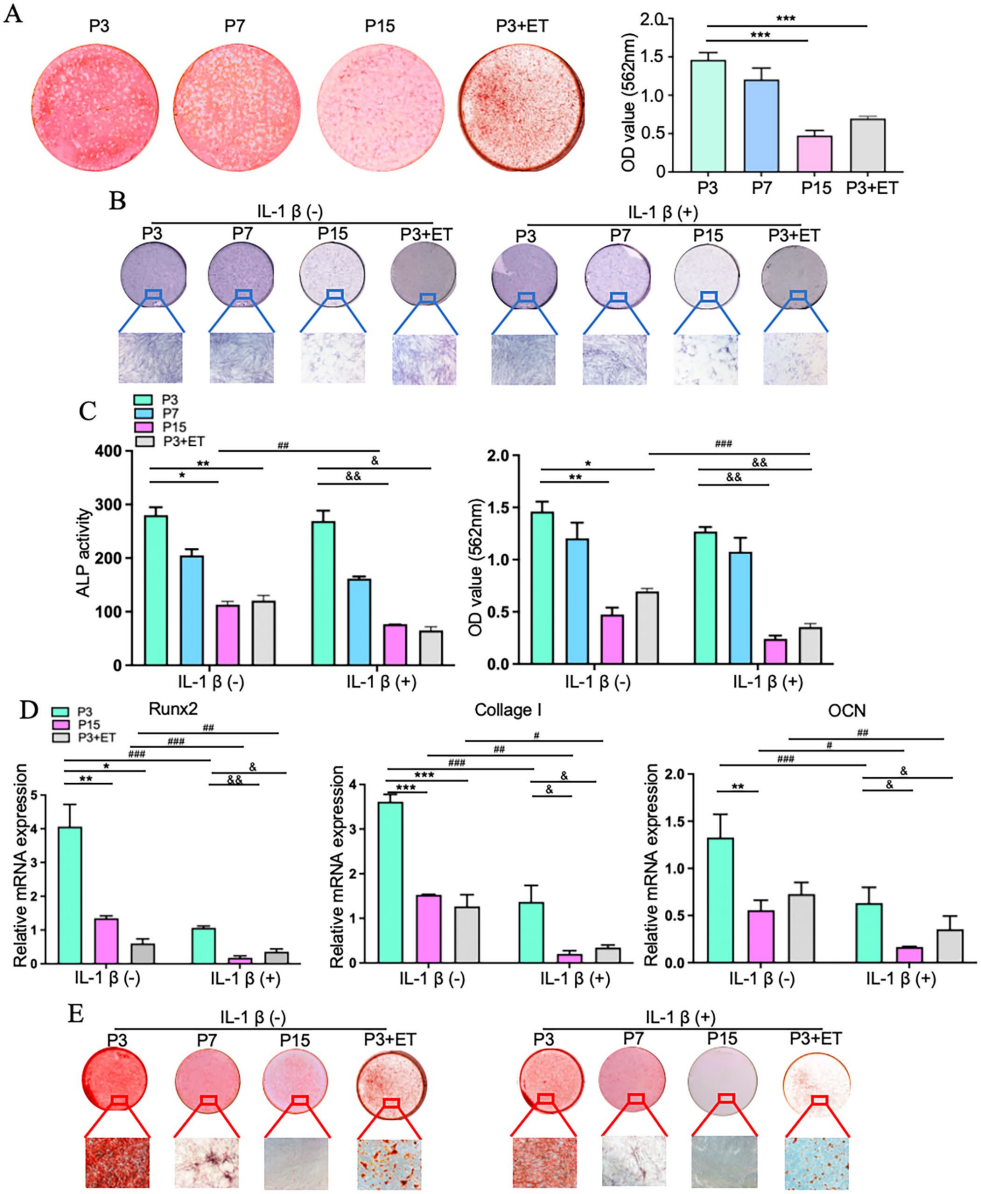
Inflammation inhibited the osteogenic differentiation capacity of aged PDLCs. (A) Mineralised nodules in P3, P7, P15, and P3 + ET PDLCs were stained with ARS following 21 days of osteogenic induction, with representative images and quantitative analysis shown. (B) ALP staining was performed on PDLCs cultured in osteogenic medium with or without IL‐1β for 7 days, shown through both macroscopic and microscopic images. (C) Quantitative evaluation of ALP activity and AR staining absorbance at 562 nm. (D) RT‐qPCR analysis of osteoblast differentiation markers (*Runx2*, *COLI*, *OCN*) in PDLCs following inflammatory stimulation. (E) ARS staining results for PDLCs cultured in osteogenic medium with or without IL‐1β for 21 days. All data are derived from three independent experiments (*n* = 3) and the number of cell donors is three (*N* = 3). Scale bar = 100 μm. Statistical significance is indicated as follows: * for differences without inflammation (*p < 0.05, **p < 0.01, ***p < 0.001); & for differences with inflammation (^&^ *p* < 0.05, ^&&^ *p* < 0.01); and # for overall differences (^#^ *p* < 0.05, ^##^ *p* < 0.01, ^###^ *p* < 0.001).

The osteogenic differentiation of P3, P7, P15, and P3 + ET PDLCs was evaluated using ARS staining after 21 days of osteogenic induction. P15 and P3 + ET PDLCs had fewer mineralised nodules than did P3 and P7 PDLCs (Figure 3A). Quantitative analysis indicated that the P15 and P3 + ET groups had lower OD values than did the other groups (Figure 3B), indicating that osteogenic differentiation decreased with ageing.

To evaluate the effects of inflammation on aged PDLCs, ALP staining was performed after 7 days of induction. The results revealed that ALP activity was decreased in aged PDLCs and declined further after IL‐1β treatment (Figure 3C). By day 21, the expression levels of osteoblast differentiation markers (*Runx2*, *COLI*, and *OCN*) were lower in aged PDLCs than in young PDLCs, particularly after IL‐1β stimulation (Figure 3D). Mineralization was also decreased in aged PDLCs, with more significant reductions observed following inflammatory stimulation (Figure 3E). Quantitatively, aged PDLCs exposed to inflammation had lower OD values than those without stimulation (Figure 3C). These results demonstrated that inflammation adversely affected the osteogenic differentiation of aged PDLCs.

### 3.4 NF‐κB/FOXO3a/c‐JUN Pathway May Be Involved in the Osteogenic Differentiation of Aged PDLCs via a Cellular Process Versus Exogenous Inflammatory Stimulation

**FIGURE 4:**
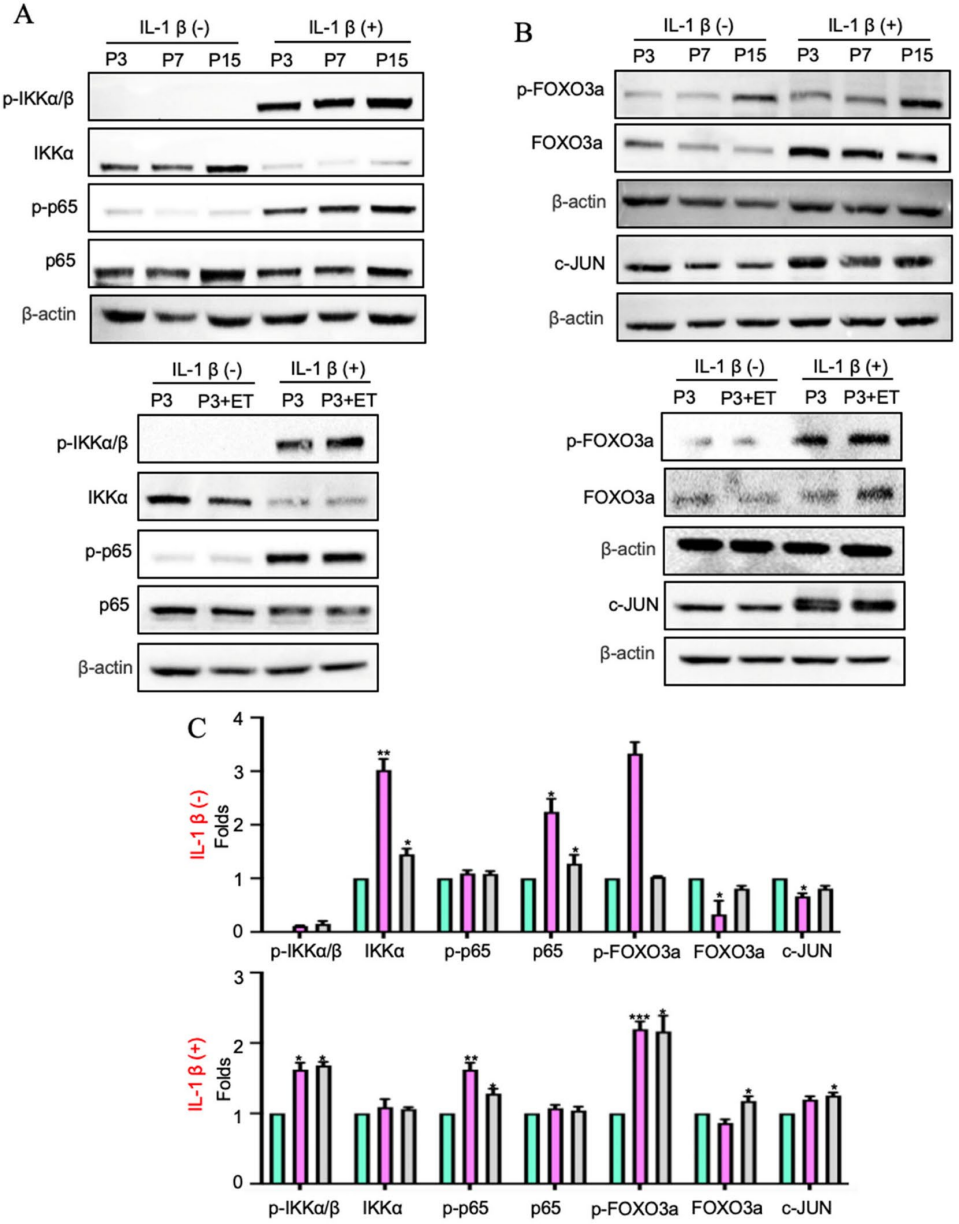
NF‐κB/FOXO3a/c‐JUN pathway may be involved in the osteogenic differentiation of aged PDLCs under cellular ageing and inflammatory stimulation. (A) Western blotting was performed to determine the expression levels of phosphorylated IKKα/β (p‐IKKα/β), IKKα, NF‐κB p‐p65, NF‐κB p65, and β‐Actin in P3, P7, P15, and P3 + ET PDLCs with or without IL‐1β stimulation. (B) Western blotting was also performed to examine the expression levels of p‐FOXO3a, FOXO3a, c‐JUN, and β‐Actin in the same groups of PDLCs with or without IL‐1β stimulation. (C) Quantitative analysis of Western blotting in p‐IKKα/β, IKKα, NF‐κB p‐p65, NF‐κB p65, p‐FOXO3a, FOXO3a and c‐JUN. All data are from three independent experiments (*n* = 3) and the number of cell donors is three (*N* = 3). Statistical significance is indicated as follows: * For differences between P15 and P3 + ET VS P3 (*p < 0.05, **p < 0.01, ***p < 0.001).

To investigate the molecular mechanisms underlying the reduction in osteogenic differentiation caused by inflammation, we examined NF‐κB‐related proteins using Western blotting (Figure 4A). Key proteins in the canonical NF‐κB pathway, including NF‐κB p65 and IKKα, exhibited increased expression in aged PDLCs (P15 and P3 + ET) compared with young PDLCs (P3 and P7, Figure 4A,C), whereas FOXO3a and c‐JUN levels were lower in aged PDLCs than in young PDLCs (Figure 4B,C). Furthermore, following IL‐1β stimulation, the expression levels of p‐IKKα/β and NF‐κB p‐p65 significantly increased (Figure 4A), which indicates that the NF‐κB pathway was activated. Meanwhile, the expression levels of p‐FOXO3a and c‐JUN increased in aged PDLCs with IL‐1β stimulation compared with cells without stimulation (Figure 4B). These findings indicated that (1) the phosphorylation of FOXO3a was activated in aged PDLCs in response to inflammatory stimulation, (2) the NF‐κB pathway was involved in regulating osteogenic differentiation in aged PDLCs under both normal and inflammatory conditions, and (3) FOXO3a and c‐JUN were critical mediators of this regulatory process.

### 3.5 Inhibition of NF‐κB Rescued the Osteogenic Differentiation of Aged PDLCs Under Inflammatory Stimulation and FOXO3a/c‐JUN Signalling Was Involved in This Process

**FIGURE 5:**
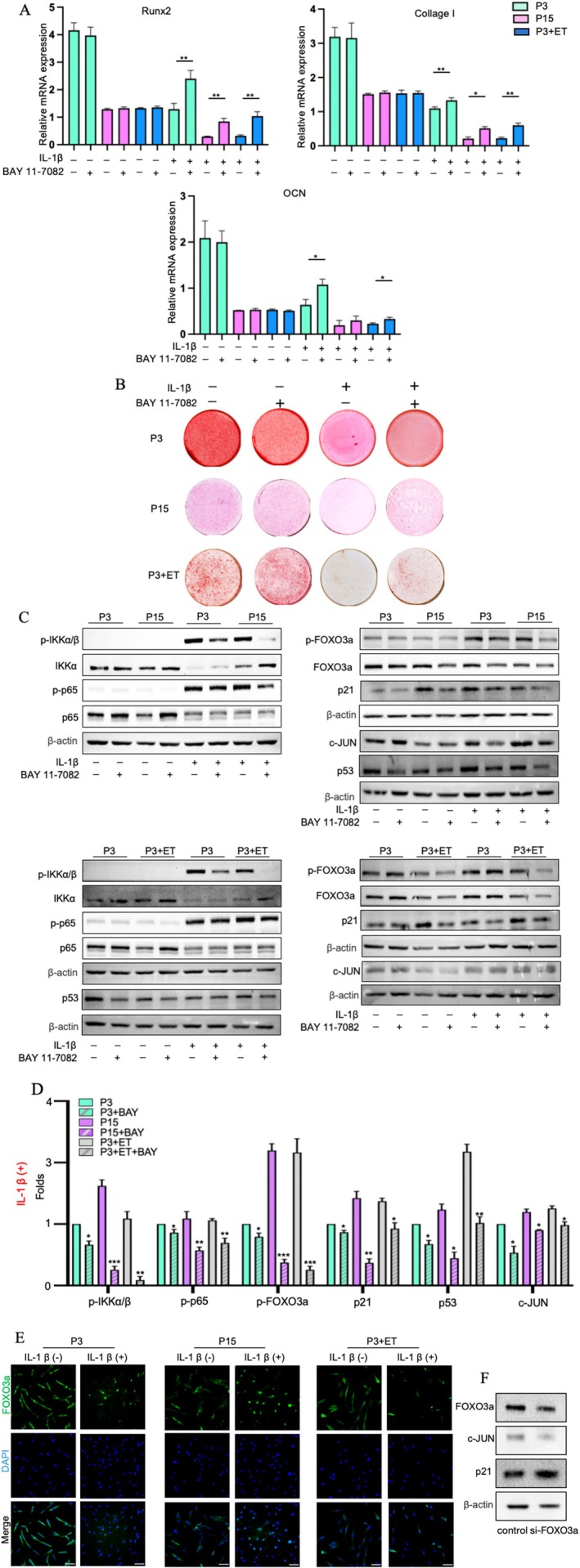
Inhibition of NF‐κB rescued osteogenic differentiation in aged PDLCs under an inflammatory microenvironment, with FOXO3a/c‐JUN signalling involved in the process. (A) mRNA levels of *Runx2, COLI*, and *OCN* were analysed using RT‐qPCR after 21 days of osteogenic induction and normalised to *β‐Actin* levels. (B) Whole‐well images of ARS staining at day 21 of osteogenic induction. (C) Western blotting was performed to assess the protein levels of p‐IKKα/β, IKKα, p‐p65, p65, FOXO3a, c‐JUN, and β‐Actin in P3, P7, P15, and P3 + ET PDLCs with or without NF‐κB inhibition. The protein levels of p53 and p21 were also examined in whole‐cell lysates through Western blotting. (D) Quantitative analysis of Western blotting in p‐IKKα/β, NF‐κB p‐p65, p‐FOXO3a, p21, p53 and c‐JUN. (E) IF staining of FOXO3a protein was performed in P3, P15, and P3 + ET PDLCs with or without IL‐1β stimulation. (F) The protein levels of c‐JUN and p21 in *FOXO3a*‐knockdown PDLCs were examined using Western blotting. All data were obtained from three independent experiments (*n* = 3) and the number of cell donors is three (*N* = 3). Scale bar = 100 μm. Statistical significance is indicated as follows: * For differences between the group with and without NF‐κB inhibition (*p < 0.05, **p < 0.01, ***p < 0.001).

To further clarify the role of NF‐κB signalling in osteogenic differentiation, we inhibited the NF‐κB signalling pathway using the NF‐κB inhibitor BAY11‐7082. The osteogenic capacity of PDLCs was then assessed using RT‐qPCR and ARS staining. As shown in Figure 5A, NF‐κB inhibition did not lead to significant differences in the mRNA expression levels of osteoblast marker genes (*Runx2*, *COLI*, *and OCN*) in PDLCs cultured under normal osteogenic conditions. By contrast, in PDLCs treated with IL‐1β, inhibition of NF‐κB resulted in the increased expression of *Runx2*, *COL1*, and *OCN*. This finding was supported by mineralization analysis (Figure 5B), which showed that blocking NF‐κB restored the formation of mineralized nodules in IL‐1β‐treated PDLCs after 21 days. These results confirm that NF‐κB signalling negatively regulates osteogenesis in aged PDLCs under inflammatory stimulation.

As shown in Figure 5C,D, inhibition of NF‐κB reduced FOXO3a phosphorylation after IL‐1β stimulation but had no significant effect on FOXO3a or c‐JUN expression in PDLCs without IL‐1β stimulation. However, blocking NF‐κB reduced p53 and p21 levels. These findings indicate that NF‐κB signalling inhibits osteoblast differentiation in aged PDLCs under inflammatory conditions by promoting *FOXO3a* phosphorylation and c‐JUN expression, with p21 exerting a synergistic inhibitory effect on osteogenesis.

To assess FOXO3a phosphorylation and localisation, we performed IF on cultured P3, P15, and P3 + ET PDLCs with and without IL‐1β stimulation (Figure 5E). In untreated PDLCs, FOXO3a was primarily localised in the cytoplasm; however, after IL‐1β stimulation, it accumulated in the nucleus. Cytoplasmic green fluorescence intensity was higher in P3 cells than in P15 and P3 + ET cells, whereas nuclear fluorescence increased in aged cells after IL‐1β stimulation. These findings are consistent with Western blotting data, confirming *FOXO3a* activation and its nuclear translocation following IL‐1β stimulation. To further explore the relationship between FOXO3a and c‐JUN, we knocked down FOXO3a and measured c‐JUN and p21 levels through Western blotting. The results revealed that *FOXO3a* knockdown reduced c‐JUN levels and increased p21 levels (Figure 5F), indicating p21 functions as a downstream effector molecule mediated by FOXO3a. Concurrently, FOXO3a also regulates c‐JUN expression.

## 4 Discussion

In this study, we found that inflammation aggravated cellular senescence and inhibited the osteogenic differentiation of aged PDLCs. During this process, NF‐κB signalling regulated osteoblast differentiation in aged PDLCs under an inflammatory microenvironment by increasing FOXO3a phosphorylation and upregulating c‐JUN expression. The inflammatory microenvironment was established by treating PDLCs with IL‐1β, a method widely adopted in recent in vitro studies of PDLCs [40]. IL‐1β is a pleiotropic cytokine and a central mediator of innate immunity and inflammation [41], and its increased level was observed at sites of periodontal damage [42].

First, our findings reveal that aged PDLCs exhibited a higher intrinsic inflammatory state than did young PDLCs and that inflammation triggered the senescence of PDLCs (Figure 2). These results are consistent with those of a previous study on osteoblasts [43], which demonstrated that exposure to an inflammatory microenvironment increased the expression of senescence markers and upregulated the expression of senescence‐associated secretory phenotype (SASP) genes. In 2000, Franceschi et al. [44] introduced the term ‘inflammageing’ to describe the progressive increase in proinflammatory status observed with ageing. This concept suggests that senescent cells accumulate with age and secrete proinflammatory molecules, creating a proinflammatory microenvironment. A previous study also observed the accumulation of senescent osteocytes in the alveolar bone of aged mice [45]. However, to the best of our knowledge, this study is the first to investigate the effect of inflammation on the senescence phenotype of PDLCs.

PDLCs are involved in the dynamic regulation of alveolar bone by mediating both bone resorption [46] and bone formation [47]. In aged patients with periodontal inflammation, alveolar bone loss is more pronounced [48]. This increased bone loss is associated with a disruption in the balance between bone formation and resorption. Therefore, understanding the osteogenic differentiation potential of PDLCs is crucial for elucidating the pathogenesis of periodontitis. Previous studies have primarily focused on osteoclastogenesis in the periodontium under inflammatory conditions [49], whereas few studies have examined the osteogenic capacity of aged PDLCs under inflammatory conditions. Inflammation is frequently associated with age‐related diseases [6] and exerts both beneficial and detrimental effects on tissue repair [50]. However, whether inflammation promotes or impairs osteogenesis in the aged periodontium and the mechanisms underlying these effects remain unclear.

To address this research gap, we examined the effect of inflammation on the osteogenic capacity of aged PDLCs by analysing mineralisation and the expression levels of osteogenesis‐related markers (*Runx2*, *COLI*, and *OCN*) in aged PDLCs (P15 and P3 + ET) under inflammatory stimulation. Our results demonstrated that inflammation negatively affected the osteogenic differentiation of aged PDLCs (Figure 3). These findings are consistent with those of previous studies indicating that osteogenic differentiation decreases in aged PDLCs and that the osteogenic potential of PDLCs is inhibited by IL‐1β stimulation [40]. However, the molecular mechanisms linking inflammation with impaired osteogenesis in ageing PDLCs were not explored in those earlier studies. *Runx2* is primarily expressed during the early stages of osteogenesis, whereas *COLI* and *OCN* are upregulated in late‐stage osteoblasts during the mineralisation phase of bone formation [51]. PDLCs comprise a heterogeneous population, including periodontal ligament stem cells, fibroblasts, alveolar bone cells, and gingival fibroblasts, which may be at different stages of differentiation and maturation. After 21 days of osteogenic induction, we observed increased expression levels of all three osteogenic markers (*Runx2*, *COLI*, and *OCN*) in PDLCs (Figure 3).

The NF‐κB signalling pathway is a canonical pathway involved in both inflammation and ageing. Under noninflammatory conditions, we found that the NF‐κB complex remained sequestered in the cytoplasm. However, upon stimulation with IL‐1β, the NF‐κB complex translocated to the nucleus (Figure 2), consistent with previous observations in lung macrophages [14] and epithelial cells [15], where inflammation was shown to induce nuclear translocation of NF‐κB. In aged PDLCs under noninflammatory conditions, the expression of SASP factors was elevated as part of the ageing response, which led to increased NF‐κB expression in the absence of phosphorylation. This was accompanied by decreased expression levels of FOXO3a and c‐JUN. Conversely, aged PDLCs under inflammatory conditions exhibited activation of the NF‐κB pathway (with phosphorylation) along with the increased phosphorylation of FOXO3a and upregulation of c‐JUN expression (Figure 4). These findings support two key conclusions. First, the NF‐κB/FOXO3a pathway is involved in regulating osteogenic differentiation in aged PDLCs under inflammatory stimulation. Second, a notable phenomenon was observed: under control conditions, osteogenic differentiation was positively correlated with c‐JUN expression levels in aged PDLCs, whereas, in an inflammatory environment, osteogenic differentiation was negatively correlated with c‐JUN expression levels. This finding indicates that although the NF‐κB/FOXO3a pathway is involved in osteogenic differentiation in both ageing and inflammation, the underlying regulatory mechanisms differ (Figure 4).

To further investigate the mechanism through which NF‐κB signalling regulates the osteogenic differentiation of aged PDLCs under inflammatory conditions, we treated the cells with an NF‐κB inhibitor that blocks NF‐κB phosphorylation. Inhibition of NF‐κB phosphorylation resulted in no significant difference in mineralization levels between young and aged PDLCs cultured in a noninflammatory medium. However, inhibition of NF‐κB phosphorylation led to an increase in the number of mineralized nodules in PDLCs exposed to inflammatory stimulation (Figure 5). Furthermore, under normal ageing conditions, we observed no differences in the levels of NF‐κB‐regulated proteins, such as IKKα and NF‐κB p65, between cells treated with the NF‐κB phosphorylation inhibitor and untreated cells (Figure 5). However, under inflammatory stimulation, the NF‐κB phosphorylation inhibitor reduced the phosphorylation of NF‐κB, leading to a decrease in the levels of phosphorylated FOXO3a, c‐JUN, and p21 (Figure 5).

Based on our aforementioned findings, we can summarise the similarities and differences in osteogenesis and regulatory mechanisms between PDLCs undergoing cellular ageing and those exposed to external inflammatory stimulation. In both conditions, osteogenic differentiation was decreased due to the inhibitory effects of ageing and inflammation. However, the molecular mechanisms underlying the involvement of NF‐κB in osteogenic differentiation differ between the normal aged periodontium and the aged periodontium exposed to external inflammatory stimuli.

First, the disparity lies in whether there is merely an upregulation of NF‐κB expression or actual activation of this signalling pathway. In cellular ageing PDLCs, the enhanced SASP response led to increased NF‐κB expression without phosphorylation, which retained the NF‐κB complex in the cytoplasm. Conversely, external inflammatory stimulation triggered the translocation of the NF‐κB complex to the nucleus and induced its phosphorylation, indicating activation of the signalling pathway. As shown in Figure 5, under normal ageing conditions, there were no observable differences in the levels of NF‐κB‐regulated proteins, such as IKKα and NF‐κB p65, between cells treated with the NF‐κB inhibitor and untreated cells because the inhibitor prevented NF‐κB phosphorylation. However, in aged cells exposed to inflammation, NF‐κB phosphorylation decreased following treatment with the inhibitor.

Second, the regulation of NF‐κB downstream proteins, such as FOXO3a, is associated with NF‐κB activation, specifically NF‐κB phosphorylation. During the cellular ageing of PDLCs, the elevated NF‐κB level inhibited FOXO3a expression. Conversely, in an external inflammatory microenvironment, NF‐κB phosphorylation led to increased FOXO3a phosphorylation. This, in turn, affected c‐JUN, because both FOXO3a and c‐JUN play essential roles in osteogenesis. FOXO3a is crucial for osteoblast differentiation and skeletal homeostasis [28, 29], whereas c‐JUN regulates the expression of genes involved in early osteoblast differentiation [32].

Third, the distinction lies in the regulation of c‐JUN between cellular ageing and an external inflammatory microenvironment. During cellular ageing, c‐JUN expression declines, negatively regulating osteogenic differentiation and reducing osteogenic capacity. However, after exposure to external inflammatory stimulation, increased FOXO3a phosphorylation leads to elevated c‐JUN expression, which contradicts the expected reduction in osteogenesis. Aged periodontal tissues also exhibit increased levels of senescence markers, such as p53 and p21, with p21 inhibiting osteoblast proliferation and differentiation [52]. Our results indicated that si‐FOXO3a reduces c‐JUN levels and increases p21 levels (Figure 5). The role of p21 in the osteogenic differentiation of aged PDLCs [18] and the possible interaction between decreased c‐JUN and elevated p21 (Figure 5) suggests that although c‐JUN is crucial in this process, it may not be the sole regulator. The combined effect of c‐JUN and p21 might be involved in the regulation of osteogenic differentiation in aged PDLCs under inflammatory conditions. Although c‐JUN was elevated, its effects may not outweigh those of p21, which actively hinders osteogenic differentiation, ultimately resulting in decreased osteogenic differentiation in aged PDLCs. c‐JUN is expressed throughout osteoblast differentiation [32]. p21 also takes part in the osteogenesis of BMSCs [53]. Our results demonstrated that p21 and c‐JUN function as the downstream effector molecules mediated by FOXO3a. In future studies, more experiments will be needed to elucidate the specific mechanism of p21 and c‐JUN in the osteogenic differentiation of aged periodontium.

Based on these findings of the study, the clinical implications involve two aspects, ageing and inflammation, respectively. Our findings in this study of cellular ageing showed that NF‐κB signalling downregulates the osteogenic differentiation of PDLCs by suppressing FOXO3a and c‐JUN, suggesting that pharmacologic inhibition of NF‐κB may represent a viable therapeutic strategy to enhance bone regeneration. This aligns with the reported pro‐osteogenic effects of NF‐κB blockade from Jun et al. [54], warranting further translational development of this therapeutic strategy. Inflammation activated NF‐κB p65 phosphorylation and inhibited NF‐κB signalling; phosphorylation increased osteogenesis in aged PDLCs. These findings, consistent with Zhao et al. [55] and Li et al. [56], suggest that inhibiting NF‐κB p65 phosphorylation may protect against bone loss and gingival inflammation. Future studies should explore pharmacological NF‐κB phosphorylation inhibition to enhance bone formation. Collectively, simultaneous suppression of NF‐κB expression and phosphorylation represents a promising therapeutic strategy for managing inflammatory diseases in aged individuals.

This study aims to investigate the effects of inflammation on the aged periodontium and explore the mechanism. The in vitro experiments offer more straightforward options for genetic modifications, including gene knockout, knock‐in, knockdown, and overexpression [57]. This capability enables a more focused investigation of underlying mechanisms. However, in vitro models lack the physiological complexity of living systems, particularly in cell interactions and microenvironment dynamics. The inclusion of in vivo animal models or 3D models would provide a more comprehensive validation of our experimental findings. Animal experiments possess better biological similarity to the real situation [58], providing preliminary insights into human biological processes. 3D cultures better mimic the natural extracellular matrix architecture, allowing for more physiologically relevant cell behavior [59]. In further studies, an in vivo animal model will be used to investigate the role of the NF‐κB signalling pathway in regulating bone metabolism in the aged periodontium. Meanwhile, to better replicate the in vivo microenvironment, we should prioritize mastering advanced 3D culture techniques.

Additionally, we used two independent models of ageing. The first was a widely used in vitro replicative senescence model [34, 35], and the second involved treating cells with ET [11, 36]. We conducted experiments using the two ageing models in parallel and obtained the same results, demonstrating the credibility and reliability of our findings. Although P15 and P3 + ET PDLCs exhibit validated senescence markers including p16, p21, and p53 upregulation, these cell senescence models cannot fully replicate the physiological complexity of aged tissues in the periodontium, such as microenvironmental dynamics and multicellular interactions. The incorporation of aged animal models will be essential in future investigations to validate our in vitro findings under physiological conditions.

This is the first study to investigate the signalling between inflammation and ageing during the osteogenic differentiation of PDLCs. Using an in vitro model, the relationships among components of the signalling pathway were systematically examined by selectively modulating individual elements, such as through *FOXO3a* gene knockdown. These findings reveal a novel inflammaging‐related regulatory mechanism and provide valuable insights into the regulation of osteogenic differentiation in aged PDLCs. Further research is warranted to explore potential strategies for increasing the osteogenic capacity of PDLCs in aged patients with periodontitis.

## 5 Conclusion

**FIGURE 6:**
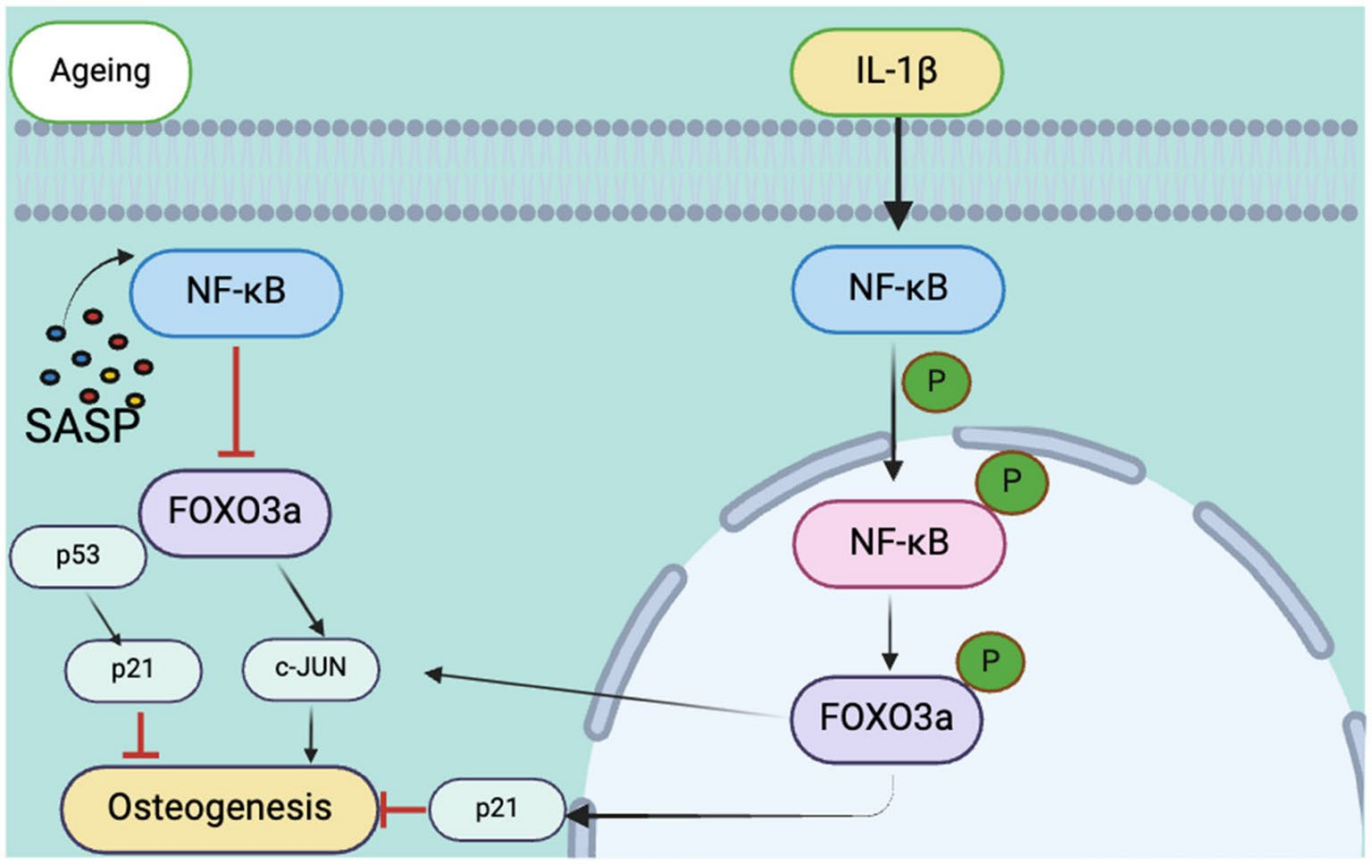
The NF‐κB/FOXO3a/c‐JUN signalling pathway plays a key role in regulating osteogenic differentiation in the aged periodontium under an inflammatory microenvironment. During the ageing process, increased levels of SASP factors lead to elevated NF‐κB expression, which in turn inhibits FOXO3a expression. Decreased FOXO3a levels result in decreased c‐JUN expression, ultimately reducing the osteogenic capacity of the aged periodontium. Upon exposure to external inflammatory stimulation, the NF‐κB pathway becomes activated, leading to NF‐κB phosphorylation and its translocation into the nucleus. NF‐κB phosphorylation leads to an increase in FOXO3a phosphorylation, resulting in increased c‐JUN expression. Moreover, the increased phosphorylation of FOXO3a triggers increased c‐JUN expression levels. In this process, senescence markers such as p53 and p21 are also upregulated, with p21 regulating osteogenesis in conjunction with c‐JUN. Elevated p21 levels further reduce the osteogenic potential of the aged periodontium under inflammatory conditions. (Created and approved by BioRender.)

As illustrated in Figure 6, cellular ageing leads to an increase in SASP factors, such as IL‐1β and IL‐6, in response to ageing [60], resulting in elevated NF‐κB expression. This increased NF‐κB expression inhibits FOXO3a expression. The consequent reduction in FOXO3a levels leads to decreased c‐JUN expression, ultimately causing a decline in the osteogenic capacity of the aged periodontium. During this process, senescence markers such as p53 and p21 are also upregulated, with p21 regulating osteogenesis in conjunction with *c‐JUN*. However, upon exposure to exogenous inflammatory stimulation, the NF‐κB pathway becomes activated, leading to NF‐κB phosphorylation and its translocation into the nucleus. NF‐κB phosphorylation triggers an increase in FOXO3a phosphorylation, which in turn elevates c‐JUN expression. In this process, increased p21 levels further inhibit the osteogenic potential of aged PDLCs, overriding the regulatory effects of c‐JUN and ultimately leading to impaired osteogenic differentiation in aged PDLCs exposed to inflammation.

## Author Contributions

L.Z. and Y.Y. designed and conducted the experiments. L.Z. and Z.T. analyzed the data, and wrote the manuscript draft. L.Z., Z.T., and X.L. initiated the project. R.H. and Y.L. edited and revised the manuscript. Y.Y. and M.G. designed the experiments and secured funding for the research. All authors have read and agreed to the published version of the manuscript.

## Disclosure

Equator Reporting Guidelines Statement: Reporting follows the CONSORT guidelines for human‐derived cell experiments.

## Ethics Statement

The experiments were approved by the Hospital Authority of Hong Kong West Cluster (reference number: UW19‐140).

## Consent

Consent was obtained for the use of anonymized patient data.

## Conflicts of Interest

The authors declare no conflicts of interest.

## Supporting information


**Figure S1:** jre70037‐sup‐0001‐FigureS1.docx.

## Data Availability

The data that support the findings of this study are available on request from the corresponding author.
