# Supplementary material for: Inflammation Affects the Osteogenic Differentiation of Aged Periodontal Ligament Cells via NF‐κB/FOXO3a/c‐JUN Signalling
Source: J Periodontal Res. 2025 Sep 17;61(3):292–308. doi: 10.1111/jre.70037 (PMC13140784; doi:10.1111/jre.70037)
Supplement: Supplementary file 1 — Figure S1: jre70037‐sup‐0001‐FigureS1.docx. [file JRE-61-292-s001.docx]

Supplemental Figure

Fig. S1.


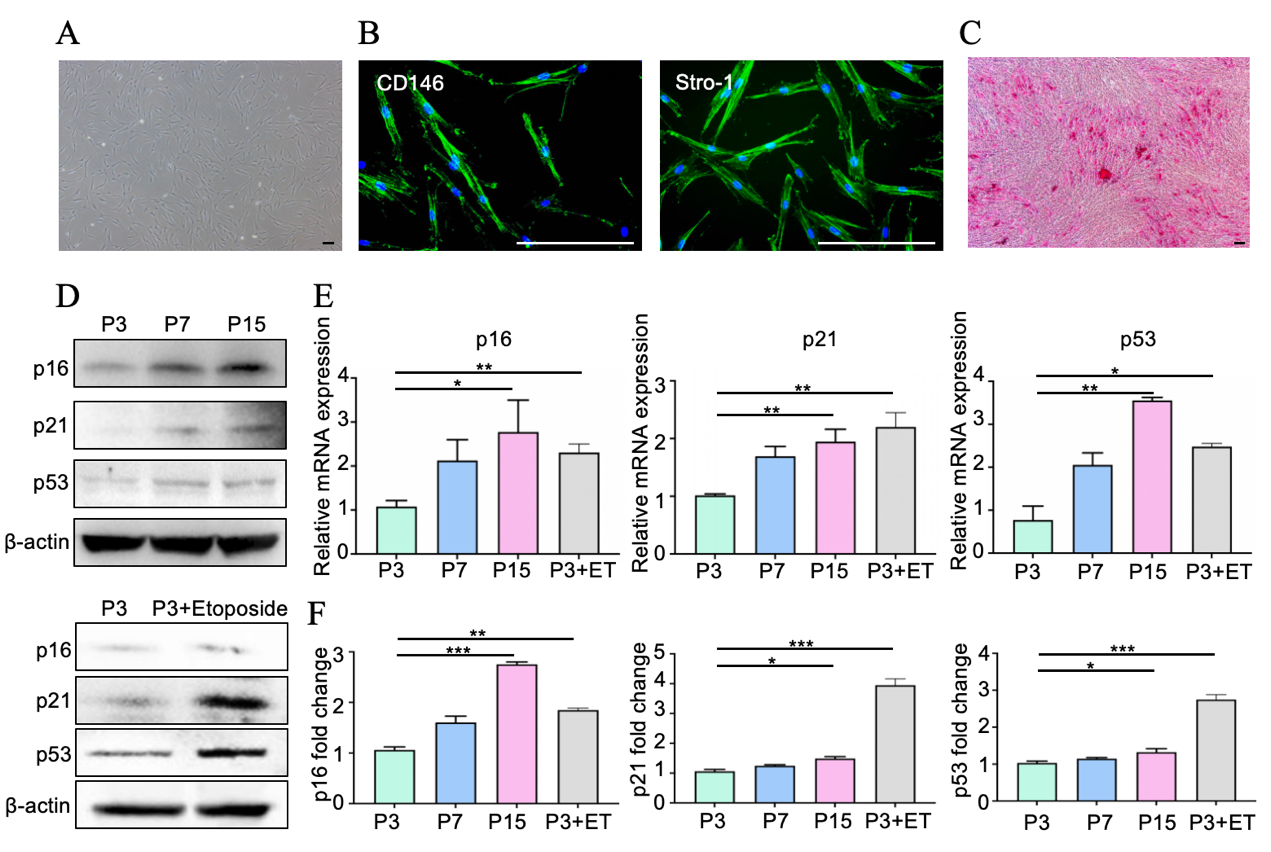


Fig. S1. Long-term passaging in ex vivo culture and etoposide treatment induced senescence in PDLCs. (A) PDLCs displayed a typical spindle-shaped morphology. (B) High expression levels of mesenchymal stem cell surface markers Stro-1 and CD146 were observed in PDLCs. (C) Mineralised nodules were detected using ARS staining following osteogenic induction. (D) Western blotting was performed to analyse p16, p21, and p53 protein levels in P3, P7, P15, and P3+ET PDLCs. (E) RT-qPCR was performed to examine p16, p21, and p15 mRNA expression in P3, P15, and P3+ET PDLCs. (F) Quantification of p16, p21, and p53 protein levels from Western blot analysis. Representative data from three independent experiments (n**=3)** and the number of cell donors is three (N=3). Scale bar = 100 μm, *p ≤ 0.05, **p ≤ 0.01, ***p ≤ 0.001).
